# Single–gene knockout of RNLS or HIVEP2 are insufficient to protect β–cell spheroids from allo– and xeno–rejection

**DOI:** 10.3389/fimmu.2026.1759835

**Published:** 2026-02-03

**Authors:** Ismail Can Karaoglu, Arda Odabas, Tamer Önder, Seda Kizilel

**Affiliations:** 1 Chemical and Biological Engineering, Koc University, Sariyer, Istanbul, Türkiye; 2 School of Medicine, Koç University, Sariyer, Istanbul, Türkiye; 3 Research Center for Translational Medicine, Koc University, Sariyer, Istanbul, Türkiye

**Keywords:** allo- and xenogeneic rejection, CRISPR, HIVEP2, RNLS, type 1 diabetes (T1D), β-cell spheroids

## Abstract

**Introduction:**

β-Cell replacement therapy offers a potential cure for type 1 diabetes, but its success is limited by rapid graft rejection. While genome-wide CRISPR screens have recently identified RNLS and HIVEP2 as candidate genes capable of protecting β-cells from autoimmune destruction, their efficacy against the distinct mechanisms of allogeneic and xenogeneic rejection remains unknown. This study aimed to test the hypothesis that single-gene ablation of RNLS or HIVEP2 protects β-cell spheroids from allo- and xenorejection in immunocompetent hosts.

**Methods:**

Murine β-TC-6 and human EndoC-βH1 β-cell lines were genetically edited using CRISPR-Cas9 to knockout RNLS or HIVEP2. Editing efficiencies were confirmed via T7 endonuclease I assay and Tracking of Indels by Decomposition (TIDE) analysis. Cells were aggregated into uniform, size-controlled spheroids using an optimized agarose suspension culture. Functional integrity was assessed via glucose-stimulated insulin secretion (GSIS). To evaluate immune evasion in vivo, luciferase-labeled spheroids were transplanted subcutaneously into immunocompetent CD-1 mice, modelling allogeneic (murine-to-murine) and xenogeneic (human-to-murine) rejection, with graft survival monitored longitudinally by bioluminescence imaging.

**Results:**

Robust editing efficiencies were achieved for both targets. Functional characterization indicated that Rnls deletion modestly impaired GSIS in murine cells, whereas HIVEP2 deletion showed no functional alterations in either cell line. In vivo assessment revealed no protective effects of RNLS or HIVEP2 deletion; grafts from both knockout groups displayed rejection kinetics indistinguishable from non-targeting controls. While allogeneic grafts survived longer than xenogeneic grafts, both were ultimately cleared by the host immune system regardless of genotype.

**Discussion:**

These data indicate that single-gene deletions of RNLS or HIVEP2 are insufficient to protect β-cell grafts from the barriers of allo- or xenorejection. By defining the limitations of these targets in isolation, our findings highlight the necessity for combinatorial genome editing strategies or complementary integration with immunomodulatory biomaterials to achieve effective and sustained β-cell graft survival.

## Introduction

Type 1 diabetes (T1D) arises from autoimmune killing of the insulin–producing β–cells of the pancreatic islets, forcing patients into lifelong exogenous–insulin therapy (1–4). This exposes them to hypoglycemia and chronic complications such as heart disease, stroke, nerve damage, and kidney failure (5–9). β–cell replacement therapy offers curative potential, yet transplanted grafts rejected rapidly to both recurrent autoimmunity and the *de novo* allogeneic or xenogeneic immune responses of the recipient. Current clinical protocols rely on broad immunosuppression, which incompletely protects grafts and carries substantial infectious and oncogenic risk (6, 10–18). Consequently, engineering β–cells that evade immune attack while preserving host immunosurveillance is a priority in the field (19–25).

Gene–editing has emerged as a powerful route to such “stealth” cells. Ablation of classical HLA class I and II molecules or over–expression of CD47 demonstrably reduces T–cell recognition, but complete removal of HLA can provoke natural–killer (NK)–cell activation and compromise pathogen defense (26–30). A complementary strategy is to identify β–cell–intrinsic genes whose loss confers resistance to immune injury without disabling antigen presentation. In this context, a genome–wide CRISPR screen performed by Cai et al. in non–obese–diabetic (NOD) β–cells uncovered two such loci, *Rnls* and *Hivep2*, whose disruption enabled mutant cells to survive autoimmune assault *in vivo* (31). *Rnls* encodes Renalase, a secreted flavoprotein oxidase that metabolizes atypical NAD(P)H isomers and has been linked to redox homeostasis and protection from cytokine–induced apoptosis. Its deletion in β–cells attenuate ER–stress signaling and dampens autoreactive CD8^+^–cell activation (32). *Hivep2* (Schnurri–2) is a zinc–finger transcription factor that binds κB motifs, modulates NF–κB–driven transcription and restrains pro–inflammatory gene expression (33). Although both knock–outs (KOs) provided an autoimmune survival advantage in the screen, their capacity to protect against the distinct mechanisms underlying allogeneic rejection (MHC mismatch and indirect antigen presentation) or xenogeneic rejection (innate immunity, complement and xeno–reactive antibodies) has never been tested.

This study aimed to evaluate the utility of RNLS and HIVEP2 beyond the autoimmune context. While RNLS served as a benchmark for platform validation in the allogeneic setting, we primarily sought to investigate the unexplored potential of these genes, particularly HIVEP2, in overcoming the more barriers of xenogeneic rejection. These genes were selected based on their previously identified roles in regulating immune-mediated cellular stress responses and apoptosis pathways. Specifically, the hypothesis here is that knocking out RNLS would protect β-cells by reducing endoplasmic reticulum stress-induced apoptosis, whereas knocking out HIVEP2 might enhance β-cell resistance by altering immune recognition and cytokine responses. *Rnls* was identified as a promising target gene in genome-wide CRISPR screening, linked primarily with apoptosis regulation under inflammatory stress conditions frequently observed in autoimmune diabetes. It was hypothesized that knocking out RNLS would mitigate stress-induced β-cell apoptosis, thus enhancing graft survival. Similarly, HIVEP2 was chosen due to its known regulatory roles in immune responses, which could protect against autoimmune attacks by modulating cytokine signaling and immune-cell interactions.

Here, it was investigated whether deleting RNLS or HIVEP2 prolongs β–cell survival beyond the autoimmune context. Murine β–TC–6 and human EndoC–βH1 cells were first edited with pLentiCRISPRv2 constructs targeting RNLS or HIVEP2, and indel formation was verified by T7 endonuclease assay and TIDE analysis. Then each line was aggregated into uniformly sized (~150 µm) spheroids on agarose–coated ultra–low–attachment dishes. Functional testing revealed that RNLS KO modestly reduced glucose–stimulated insulin secretion in mouse cells, whereas HIVEP2 KO was functionally neutral in both species. To assess graft fate in immunocompetent hosts, luciferase–labelled spheroids were embedded in Matrigel and implanted subcutaneously into CD–1 mice, modelling either allogeneic rejection (β–TC–6 → CD–1) or xenogeneic rejection (EndoC–βH1 → CD–1). Longitudinal bioluminescence imaging demonstrated indistinguishable clearance kinetics among RNLS–, HIVEP2– and non–targeting grafts in both settings. Our findings therefore show that single–gene disruption of RNLS or HIVEP2 is insufficient to protect β–cell grafts from allo– or xeno–immunity. These results set practical limits on the utility of these loci as single-gene edits and underlining the need for combinatorial genome engineering or complementary immunomodulatory biomaterials to achieve clinically durable immune evasion.

## Methods

### Cell lines

β–TC–6 and 293T (CRL–3605 and CRL–3216) cell lines were obtained from ATCC, EndoC–βH1 cell line was obtained from Human Cell Designer. 293T cells were maintained in DMEM (D6429, Sigma), supplemented with 10% FBS (S1520, Biowest), and penicillin/streptomycin (L0022, Biowest) in a 37°C incubator with 5% CO2. β–TC–6 cells were maintained in low bicarbonate DMEM (P04–03596, PAN Biotech), supplemented with 15% FBS (S1520, Biowest), and penicillin/streptomycin (L0022, Biowest) in a 37°C incubator with 5% CO2. EndoC–βH1 cells were maintained in Opti–β1 (Human Cell Design) after coating with βcoat (Human Cell Design) in a 37°C incubator with 5% CO2. All cell lines were routinely tested for mycoplasma contamination on a weekly basis and confirmed to be negative.

### gRNA cloning

To generate NT, *Hivep2* KO, *Ins2* KO, and *Rnls* KO β–TC–6 cells, three gRNAs were selected from Brie mouse gRNA database (**Supplementary Table 1**) (34) and cloned into pLentiCRISPRv2 vector. After an initial screen of T7EI and TIDE analysis, one gRNA was selected for each gene as follows: NT gRNA (5′–GCGCTTCCGCGGCCCG–3′), *Hivep2* gRNA (5′–TAAGGCGGATGACTCTCACA–3′), *Ins2* gRNA (5′–GGACTCCCAGAGGAAGAGCA–3′), and *Rnls* gRNA (5′– CTACTCCTCTCGCTATGCTC–3′) were cloned into pLentiCRISPRv2 vector with puromycin antibiotic resistance gene (Addgene #98290) (35). The gRNA containing lentiviruses were used to establish these cell lines.

To generate NT, *HIVEP2* KO, *INS* KO, and *RNLS* KO EndoC–βH1 cells, similarly, three gRNAs were selected from Brunello human gRNA database (**Supplementary Table 1**) (34) and cloned into pLentiCRISPRv2 vector. After an initial screen of T7EI and TIDE analysis, one gRNA was selected for each gene as follows: NT gRNA (5′–GACGGAGGCTAAGCGT–3′), *HIVEP2* gRNA (5′–GACAAGATGTCAGACCTAGG–3′), *INS* gRNA (5′–GAAGCTCTCTACCTAGTGTG–3′), and *RNLS* gRNA (5′–TCCCACACAGCAAGGTACAA–3′) were cloned into pLentiCRISPRv2 vector with puromycin antibiotic resistance gene. All plasmid sequences were verified by Sanger sequencing before transduction and transfection (**Supplementary Figure 1**).

### Lentivirus production

293T cells were seeded at 4.0 × 106 cells on a 10–cm plate 1 day before transfection. The cells were transfected with 2.5 μg of a vector, 2.25 μg of a transfer plasmid (psPAX2, #12260; Addgene), and 0.25 μg of a VSV–G vector (pMD2.G, #12259; Addgene) using the polyethylene imine (PEI) transfection reagent (Thermo Fisher Scientific, Waltham, MA). Viral particles were harvested 48 h post transfection and purified using a 0.45–μm Millex–HV filter (SLZHVR33RS; Merck Millipore, Burlington, MA).

### Virus infection and drug selection

For viral transduction, 5 × 10^6^ cells were seeded in 10–cm tissue–culture dishes and allowed to attach for 24 h. The cultures were then exposed overnight to 10 µL of 100–fold–concentrated lentiviral stock supplemented with 10 µg/mL protamine sulfate (P3369, Sigma–Aldrich) to enhance viral adsorption. Fresh growth medium was provided the next morning. Forty–eight hours later, robust fluorescence in parallel GFP–virus controls confirmed successful transduction, and antibiotic selection was initiated. β–TC–6 cultures received 4.5 µg/mL puromycin, whereas EndoC–βH1 cultures were treated with 2.5 µg/mL. Selection was maintained for four days, after which surviving cells were expanded for an additional two days in antibiotic–free medium to recover. All downstream assays were performed with these fully selected, puromycin–resistant populations.

### T7 endonuclease I assay

CRISPR–Cas9 editing in both β–TC–6 and EndoC–βH1 cells were detected by T7 endonuclease I mismatch cleavage assay. Genomic DNA was purified from NT and gRNA transduced cells using the Macherey–Nagel™ NucleoSpin™ Tissue, Mini kit for DNA from cells and tissue. The Hivep2, Rnls, and insulin gRNA targeting site was amplified using the Phusion High–Fidelity PCR Kit. The primers for the gRNA site PCR were as follows: *Hivep2* site forward, 5′–CTCCCTGCTGAGAAGTTGCC–3′; reverse, 5′– GATGTGGCTGTTCGGGTAAG –3′, *Ins2* site forward, 5′–CGTGAAGTGGAGGACCCACA–3′; reverse, 5′–AAACTGTGGGTCCTCCACTTCACG–3′, *Rnls* site forward, 5′–TATCCCATGTGGCTTGGAGT–3′; reverse, 5′–GAGGTGAAGAATCGGTCCACT–3′, *HIVEP2* site forward, 5′–GTTGTGTTGCCGAACTAGCC–3′; reverse, 5′–GGGGACAGGGTTGGGTATGA–3′, *INS* site forward, 5′–CCACCCTCTGATGTATCTCGG–3′; reverse, 5′–AGACTATAAAGCCAGCGGGG–3′, *RNLS* site forward, 5′–GAGTGGAATCAATCTTAGCAGTTG–3′; reverse, 5′–GAGTGGAATCAATCTTAGCAGTTG–3′. The 400 ng PCR products were used to form heteroduplexes by denaturing at 95°C for 5 min and then reannealing the products in a thermocycler using the following protocol: ramp down to 85°C at −2°C per s; ramp down to 25°C at −0.1°C per s; hold at 4°C. Ten units of T7 endonuclease I (#M0302) were added to the annealed PCR products and the reaction was incubated at 37°C for 15 min. The digestion reaction was stopped by 1 μl of 0.5 M EDTA and immediately applied to a 1% agarose gel to visualize digested and undigested products by electrophoresis.

### quantitative PCR

β–TC–6 and EndoC–βH1 cells were treated with Macherey–Nagel™ NucleoSpin™ RNA, Mini kit for RNA purification for RNA extraction according to the manufacturer’s protocol. Purified RNA was reverse transcribed into complementary DNA (cDNA) using the iScript™ cDNA Synthesis Kit. Gene expression levels of genes were analyzed by QuantiNova Probe PCR Kit (Qiagen). The primers for the qPCR were as follows: Hivep2 site forward, 5′–GTTGAGTCTTGGACATTGAAGC–3′; reverse, 5′–CACAGGACATCAGAATACTACTGG–3′, Ins2 site forward, 5′–CGTGGCTTCTTCTACACACCCA–3′; reverse, 5′–TCCAGTGCCAAGGTCTGAAGGT–3′, Rnls site forward, 5′–AAGGCTGGGGATATAGGGGG–3′; reverse, 5′–GGGAGACTTCTGCACCTGAC–3′, HIVEP2 site forward, 5′–ACAGCCACTAGATTCCCAGA–3′; reverse, 5′–TTGTCCTGACGCTTCCTGTT–3′, INS site forward, 5′–AGGCCATCAAGCAGATCACT–3′; reverse, 5′–TTCCCCGCACACTAGGTAGA–3′, RNLS site forward, 5′–GGGAGCACGGAACCAAAGA–3′; reverse, 5′–AGTCATTCTTCCCCCTGAGTC–3′. The relative mRNA expression levels were normalized to GAPDH mRNA expression. All RT–qPCR assays were performed using a LightCycler^®^ 480 Instrument II (Roche Diagnostics).

### Hanging–drop aggregation

Single–cell suspensions were prepared in complete growth medium (DMEM, 10% FBS, 1% penicillin/streptomycin) at 1 × 10^7^ cells/mL. Thirty–microliter droplets containing either 300, 500, or 1000 cells were dispensed onto the inner surface of 100–mm Petri–dish lids. Lids were inverted over dishes containing 10 mL sterile PBS to maintain humidity and incubated for 72 h at 37°C, 5% CO_2_. Spheroids that formed at the bottom of the droplet were harvested by gentle pipetting with pre–warmed medium.

### Home-made ultra–low–attachment (ULA) suspension culture

For higher throughput, homemade ULA dishes were fabricated by pouring 5 mL of sterile 1% (w/v) agarose in PBS into 100–mm Petri dishes, allowing the gel to solidify for 30 min, and rinsing once with growth medium. Subsequently, 5 × 10^6^ single cells in 10 mL medium were added per dish and cultured on an orbital shaker (50 rpm) for 3 days at 37°C, 5% CO_2_. Gentle agitation promoted rapid self–assembly into compact spheroids with minimal size heterogeneity.

### Live/dead staining and imaging

Spheroids from both protocols were washed twice with warm PBS and incubated for 3 min with fluorescein diacetate (FDA, 2 µg/mL) at 37°C to label metabolically active cells. Propidium iodide (PI, 1 µg/mL) was then added for an additional 5 min to stain membrane–compromised cells. After two PBS washes, spheroids were transferred to glass–bottom dishes and imaged immediately by epifluorescence microscopy (FITC and Texas Red filter sets). Aggregate diameters were measured from bright–field micrographs using ImageJ.

### Immunofluorescence staining

Immunofluorescence was performed to assess insulin production in both 2D monolayer cultures and 3D spheroids. For 2D cultures, EndoC-βH1 cells were fixed 72 h after seeding. For 3D cultures, spheroids were harvested by centrifugation at 100 × g for 5 min and the resulting pellets were fixed with 4% paraformaldehyde (PFA) for 15 min. Following fixation and washing with PBS, samples were permeabilized with 0.1% Triton X-100 and blocked with bovine serum albumin (BSA) to prevent non-specific binding. Samples were incubated overnight at 4°C with a polyclonal guinea pig anti-insulin primary antibody (1:50; PA1-26938, Thermo Fisher Scientific). After washing, samples were incubated with an Alexa Fluor 488-conjugated anti-guinea pig secondary antibody (1:250; ab150185, Abcam) for overnight at room temperature. Nuclei were counterstained with DAPI (1:5000; D1306, Thermo Fisher Scientific). Spheroids were transferred to 8-well chambered glass slides and imaged using a confocal laser scanning microscope.

### Glucose stimulated insulin secretion

For glucose–stimulated insulin secretion assay, the cells were plated in a 6–well plate as 1.5 × 106 cells per well. After 48h, cells were washed twice very gently using Krebs–Ringer Buffer (KRB). Before starting GSIS, cells were starved for 60 minutes by leaving cells in 2 mL of KRB buffer and incubating at 37°C, 5% CO2. After starvation, KRB buffer was removed and cells were incubated using 2.8 mM D–Glucose KRB (low glucose, LG) at 37°C, 5% CO2 for 60 minutes. Supernatant was collected and then cells were finally incubated using 16.7 mM D–glucose KRB (high glucose, HG) for 60 minutes at 37°C, 5% CO2, and the supernatant was collected. Supernatant was used to perform insulin quantification using the insulin ELISA kit from Mercodia.

For β–TC–6 the KRB contained (in mmol/L): 115 NaCl, 5.3 KCl, 2.6 CaCl_2_, 1.2 MgSO_4_, 1.0 KH_2_PO_4_, 24 NaHCO_3_ and 10 HEPES, supplemented with 0.1% (w/v) fatty–acid–free BSA and either 1.67 mM (LG) or 16.7 mM (HG) glucose. The solution was equilibrated in 5% CO_2_ to maintain pH 7.35–7.40.

For EndoC–βH1 the KRB containing (in mmol/L): 138 NaCl, 5.4 KCl, 2.6 CaCl_2_, 0.5 MgCl_2_, 5 NaHCO_3_ and 10 HEPES, again with 0.1% fatty–acid–free BSA and either 2.8 mM (LG) or 28 mM (HG) glucose.

### *In vivo* imaging

To enable *in vivo* bioluminescence tracking, both β–cell lines were first transduced with a lentiviral construct with the firefly luciferase (Fluc). Fluc coding sequence is driven by the human ubiquitin–C (ubC) promoter with a selection marker of Neomycin (G418) or hygromycin B (**Supplementary Figure 14**). Stable cell lines were obtained by antibiotic selection, 400 µg/mL G418 for β–TC–6 cells and 200 µg/mL hygromycin B for EndoC–βH1 cells, resulting in uniform Fluc expression. To ensure absolute parity in reporter expression across experimental groups, all gene-edited lines were generated from the same parental stable luciferase-expressing cell pool, thereby ensuring an identical reporter pedigree prior to CRISPR-Cas9 editing.

For *in vivo* studies, 5 × 10^6^ Fluc–cells from each knockout line, together with a NT control, were resuspended in 200 µL Matrigel (CLS354234, Merck) and injected subcutaneously into the left and right flank of CD–1 mice (designated Day 0). Bioluminescence imaging (BLI) was performed with a Caliper IVIS Spectrum system (PerkinElmer) following established protocols for the longitudinal monitoring of β-cell graft survival in murine models (36). Ten minutes before image acquisition, animals received an intraperitoneal injection of d–luciferin (150 mg/kg of animal; P1042, Promega) prepared at 15 mg/mL in calcium– and magnesium–free Dulbecco’s PBS and sterile–filtered through a 0.22–µm membrane. Imaging parameters were kept constant throughout the study (exposure = 1 min, binning = medium, f/stop = 1, field of view = 12.5 cm) to permit quantitative comparison. Photons emitted from the graft region were integrated with Living Image software and expressed as radiance (photons/scm²sr).

### Animal experiments

All *in vivo* procedures were approved by the Koç University Animal Research Facility Ethical Committee (KUARF, approval no. 2024.HADYEK.038) and conducted in accordance with the ARRIVE guidelines and Turkish Animal Welfare regulations. Female CD–1 or SCID mice (8–10 weeks, 25–30 g) were housed in individually ventilated cages (22 ± 2°C, 50 ± 10% humidity, 12 h light/dark cycle) with ad libitum access to standard chow and water.

For each genetic condition, NT control, *Hivep2/HIVEP2* knockout, *Rnls/RNLS* knockout, and *Ins2/INS* knockout, n = 4 mice were used in both the allogeneic (β–TC–6 → CD–1) and xenogeneic (EndoC–βH1 → CD–1) cohorts. On day 0 mice were anesthetized with 2% isoflurane, and spheroids from 5 × 10^6^ luciferase–expressing cells suspended in 200 µL growth–factor–reduced Matrigel were injected subcutaneously into each flank (left, NT; right, gene–edited) and visualized via IVIS. Mice were euthanized by CO_2_ inhalation followed by cervical dislocation.

### Statistical analysis

One–way ANOVA with Tukey comparison test on GraphPad Prism Software (Graph–Pad Software version 10.5, CA, USA) was used. A value of p ≤ 0.05 was considered statistically significant. The significance levels are denoted as follows: n.s. for p > 0.05, * for p ≤ 0.05, ** for p ≤ 0.01, *** for p ≤ 0.001, and **** for p ≤ 0.0001.

## Results

### β–TC–6 and EndoC–βH1 cell spheroid optimization

**Figure 1:**
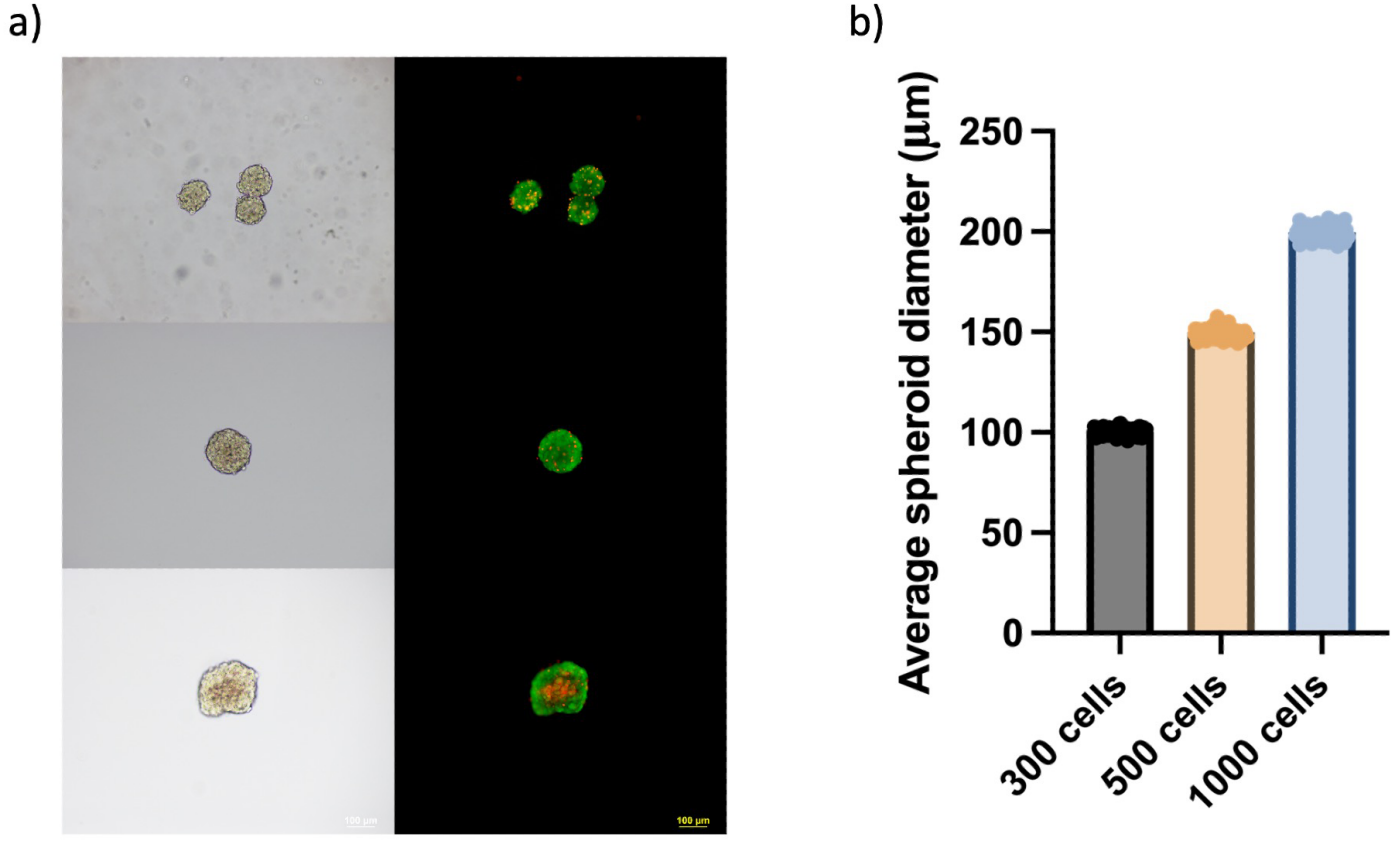
Hanging–drop optimization of β–TC–6 spheroid size and viability. **(a)** Representative bright–field (left) and Live/Dead (right; fluorescein diacetate, green = viable; propidium iodide, red = non–viable) images of spheroids generated from 300, 500 and 1000 cells per 30 µL hanging drop (top to bottom). Spheroids seeded with 1–000 cells routinely exceed 200 µm and display a central necrotic core, whereas aggregates formed from ≤ 500 cells remain compact and uniformly viable. (Scale bar = 100 µm) **(b)** Quantification of average spheroid diameter (mean ± SD, n = 100 independent droplet sets per condition). Diameter increases proportionally with initial cell number, emphasizing the need to limit seeding density to < 500 cells to maintain physiologically relevant (< 150 µm), necrosis–free aggregates.

Prior to the gene–editing experiments, 3D β–cell culture was optimized to obtain size–controlled, high–viability spheroids that more closely mimic the cell–cell interactions of native islets. The first approach relied on the hanging–drop technique. Single–cell suspensions of β–TC–6 cells were adjusted to 300, 500, or 1000 cells per 30 µL droplet, arrayed on the inverted lids of Petri dishes and cultured for 72 h at 37°C under 5% CO_2_. During this period, cells settle at the bottom of the droplet due to gravity, where they aggregate into spheroids. Bright–field microscopy revealed a direct relationship between initial seeding density and final aggregate size. Droplets seeded with 300 cells produced spheroids averaging 120 µm in diameter, whereas those initiated with 1000 cells routinely exceeded 200 µm. Live/Dead staining demonstrated that aggregates below 150 µm were uniformly viable, while the larger spheroids displayed a central necrotic zone, consistent with oxygen– and nutrient–diffusion limitations (**Figures 1A, B**) (37). Although the method can be considered as simple, the hanging–drop format proved labor–intensive, and difficult to scale beyond a few hundred spheroids, making it impractical for the cell quantities required for transplantation.

**Figure 2:**
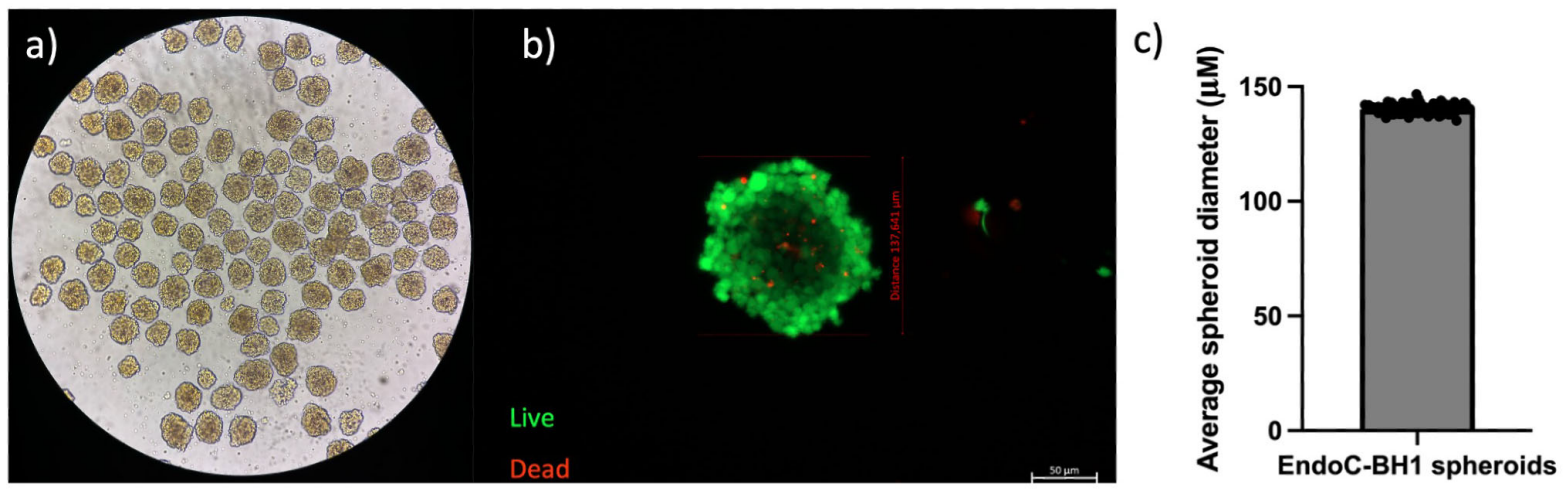
Suspension–culture generation of uniformly sized, viable EndoC–βH1 spheroids. **(a)** Bright–field overview of the agarose–coated ultra–low–attachment dish after 72 h of orbital shaking (50 rpm) with 5 × 10^6^ human EndoC–βH1 cells, showing hundreds of compact aggregates. **(b)** Live/Dead confocal image of a representative spheroid stained with fluorescein diacetate (green, viable) and propidium iodide (red, non–viable). The absence of red signal confirms minimal core necrosis. Scale bar = 50 µm. **(c)** Mean spheroid diameter determined from 100 aggregates (140 ± 8 µm; mean ± SD), illustrating the narrow size distribution achieved with the shaking suspension method.

To overcome these constraints, a suspension culture protocol was developed using home–made ultra–low–attachment dishes. Petri dishes were coated with 1% (w/v) agarose solution and allowed to solidify, creating a non–adhesive surface that prevents cell attachment. 4 × 10^6^ single cells were added to each dish in 8 mL of complete medium and cultured for 72 h on an orbital shaker set to 50 rpm. Under these conditions, cells aggregated spontaneously into hundreds of uniformly round spheroids (**Figures 2A, B**). Image analysis of 100 aggregates showed a narrow size distribution centered at 140 ± 8 µm, while confocal Live/Dead staining confirmed ≥ 95% viability throughout the spheroid cross–section (**Figure 2C**). Notably, the suspension approach yielded a higher number of spheroids per dish compared to the hanging–drop method, making it the preferred method for downstream experiments [Costa et al., 2018].

Given its superior throughput, precise size control, and absence of a necrotic core, the agarose–based suspension culture was selected for all subsequent CRISPR/Cas9 editing, functional assays, and transplantation studies. While the hanging–drop data provided a valuable reference for correlating spheroid diameter with diffusion limits, this information guided our decision to maintain aggregate diameters below 150 µm in all subsequent experiments.

### T7 endonuclease I and tracking of indels by decomposition analysis

To perform targeted gene knockouts, three guide RNAs (gRNA) were selected from the Brie and Brunello databases, including the guide RNA previously validated by Cai et al. as a control for Rnls knockout (31). The aim was to target exons as early as possible to maximize the probability of premature stop codons triggering nonsense-mediated decay (NMD) or producing truncated non-functional protein fragments, ensuring the disruption of the gene’s biological activity. As an internal control for functional knockout validation, the insulin gene (*Ins2* for mouse, *INS* for human) was targeted in both cell lines. Each selected guide RNA was cloned into the pLentiCRISPRv2_puro plasmid, and successful insertion was confirmed by Sanger sequencing. Lentiviral particles were then produced by co–transfection of HEK293T cells with the guide RNA–cloned pLentiCRISPRv2_puro plasmids along with packaging (psPAX2) and envelope (VSV–g) plasmids. GFP–encoding lentivirus was produced similarly and used as a control to monitor infection efficiency. Following lentiviral production, β–TC–6 and EndoC–βH1 cell lines were infected with lentiviruses encoding gRNAs targeting RNLS, HIVEP2, and INS. After antibiotic selection to enrich for successfully transduced cells, cell pellets were collected and used for subsequent validation assays, including quantitative PCR (qPCR), genomic DNA isolation for T7 endonuclease I (T7E1), Tracking of Indels by Decomposition (TIDE) assays to confirm successful genomic editing at the intended target sites. TIDE analysis was specifically employed to quantify the ratio of frame-shift mutations, ensuring that the edited populations were dominated by out-of-frame indels that preclude the formation of functional proteins.

**Figure 3:**
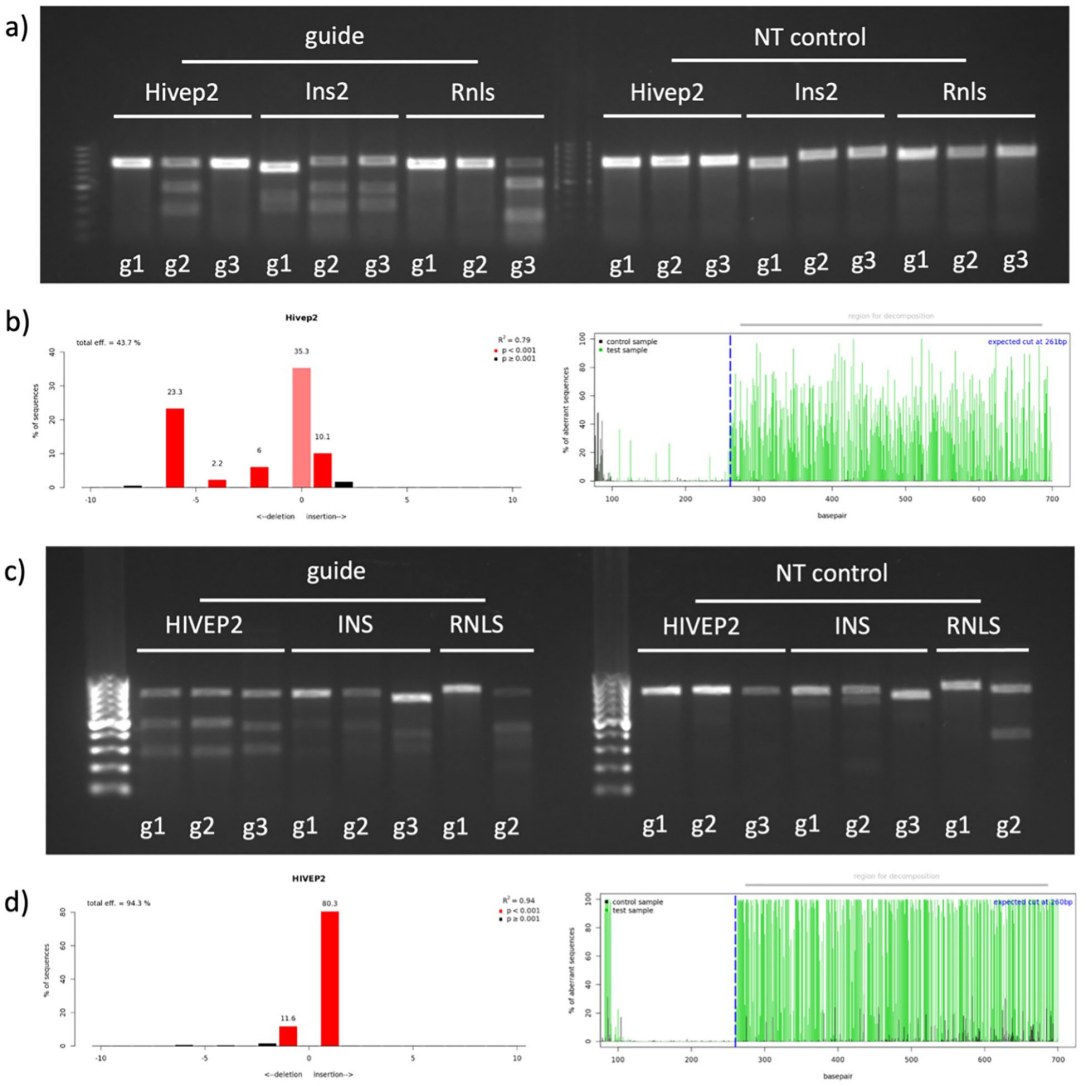
Genomic validation of CRISPR/Cas9 editing in mouse and human β–cell lines. **(a)** T7E1 assay of genomic PCR products amplified from stable, antibiotic-selected populations of β–TC–6 cells with three gRNAs targeting *Hivep2*, *Ins2* or *Rnls* (left), alongside the corresponding non–targeting (NT) controls (right). Cleavage products visible for *Hivep2*–g2, all three *Ins2* guides and *Rnls*–g3 confirm formation of indels, whereas NT lanes show only full–length amplicons. **(b)** TIDE quantification in mouse cells. Left: indel percentage. Right: representative TIDE decomposition. **(c)** T7E1 assay of genomic PCR products amplified from stable, antibiotic-selected populations of EndoC–βH1 cells edited with guides against *HIVEP2*, *INS* or *RNLS*. Cleavage is evident for all three *HIVEP2* and *INS* guides, and for *RNLS*–g2, whereas NT controls are uncleaved. **(d)** TIDE quantification in human cells. Left: indel percentage. Right: representative TIDE decomposition.

To validate successful genome editing in β–TC–6 cells, T7E1 mismatch assays were conducted for the *Hivep2*, *Ins2*, and *Rnls* gene targets. Distinct cleavage products were observed for gRNA2 targeting *Hivep2*, all three gRNAs targeting *Ins2*, and gRNA3 targeting *Rnls*, confirming the presence of CRISPR/Cas9–induced insertions and deletions (indels). No cleavage bands were detected in the corresponding non–targeting (NT) control lanes, confirming editing specificity (**Figure 3A**). Consistent with the T7E1 results, TIDE analysis of *Hivep2*–edited β–TC–6 cells using gRNA2 revealed editing efficiency of approximately 43.7%, further validating robust gene disruption at the target site (**Figure 3B**).

In parallel, genome editing in EndoC–βH1 cells was assessed using the same approach. T7E1 assays revealed clear cleavage products for all three gRNAs targeting *HIVEP2*, *INS*, and gRNA2 targeting *RNLS*, indicating efficient CRISPR–mediated gene knockout events (**Figure 3C**). TIDE analysis of the *HIVEP2* locus edited with gRNA1 demonstrated a high indel frequency of approximately 94.3%, corroborating the T7E1 data and confirming moderate editing efficiency in human β–cells (**Figure 3D**).

To evaluate whether these high-frequency genomic indels resulted in corresponding changes at the transcript level, we conducted quantitative PCR (qPCR) analysis for the targeted loci (**Supplementary Figure 2**). The resulting data revealed a complex transcriptional landscape consistent with the phenomena of transcriptional adaptation or genetic compensation. While *Rnls* knockout in murine cells led to a significant reduction in mRNA levels, likely reflecting nonsense-mediated decay (NMD)-mediated transcript instability (38), we observed an unexpected upregulation of *Hivep2* mRNA levels in murine cells. Such outcomes are indicative of cellular attempts to maintain genetic robustness by upregulating a mutant locus or its functional homologs following the loss of a functional protein product, even when the transcripts carry frame-shift mutations that preclude functional protein synthesis. Given the potential for these complex feedback loops to confound mRNA quantification, we prioritized high-resolution genomic indel analysis and functional physiological assays as the most definitive evidence of successful gene inactivation in this study.

### Insulin production and glucose stimulated insulin secretion of human β–cells

#### Insulin granules in monolayer EndoC–βH1 cells

**Figure 4:**
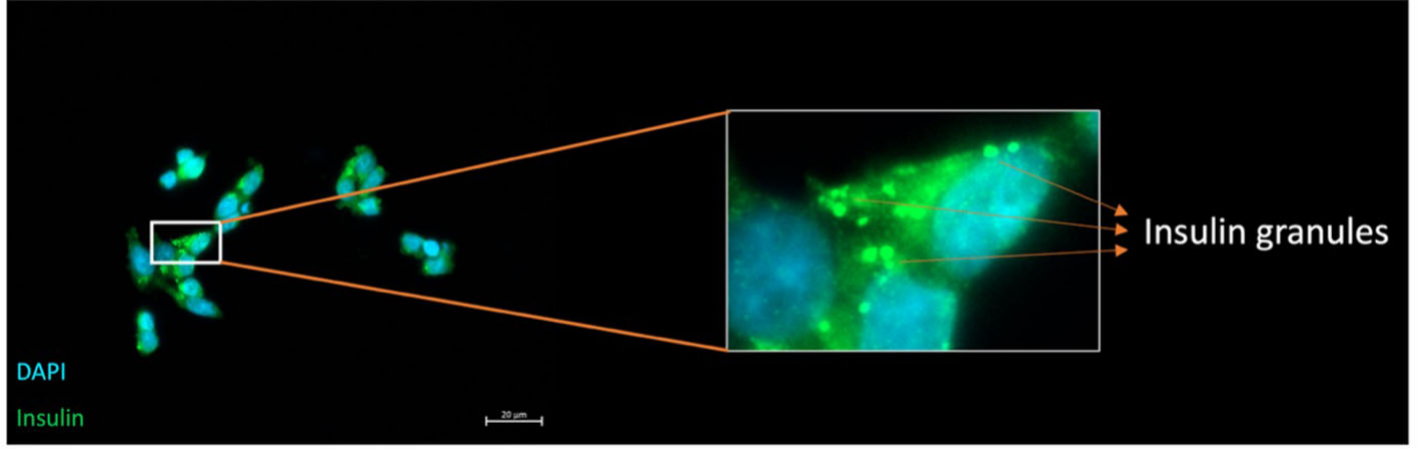
Immunofluorescence demonstration of insulin–containing granules in EndoC–βH1 cells. Representative confocal micrograph of unedited EndoC–βH1 cells cultured as a monolayer.  Insulin immunoreactivity (green) appears as concentrated in the perinuclear region, while nuclei are counter–stained with DAPI (blue).  The magnified inset highlights individual dense–core insulin granules (arrowheads), confirming intact endocrine granule biogenesis in the human β–cell line. (Scale bar = 20 μm)

To verify functionality of human β–cell line, parental EndoC–βH1 cells was grown in two–dimensional monolayer by immunofluorescence microscopy (**Figure 4**). Cells were fixed 72 h after seeding, permeabilized, and stained for insulin, with nuclei counter–labelled using DAPI. Confocal imaging revealed intense insulin signal concentrated in the perinuclear cytoplasm of every cell, consistent with dense–core secretory granules typical of mature β–cells. The presence of discrete green granules confirms preserved pro–insulin processing and granule biogenesis which is prerequisite for physiologically relevant glucose–stimulated insulin secretion and validates EndoC–βH1 as an appropriate human model for downstream gene–editing and transplantation studies.

Representative confocal micrograph of unedited EndoC–βH1 cells cultured as a monolayer. Insulin immunoreactivity (green) appears as concentrated in the perinuclear region, while nuclei are counter–stained with DAPI (blue). The magnified inset highlights individual dense–core insulin granules (arrowheads), confirming intact endocrine granule biogenesis in the human β–cell line. (Scale bar = 20 µm).

#### Insulin production in EndoC–βH1 spheroids

**Figure 5:**
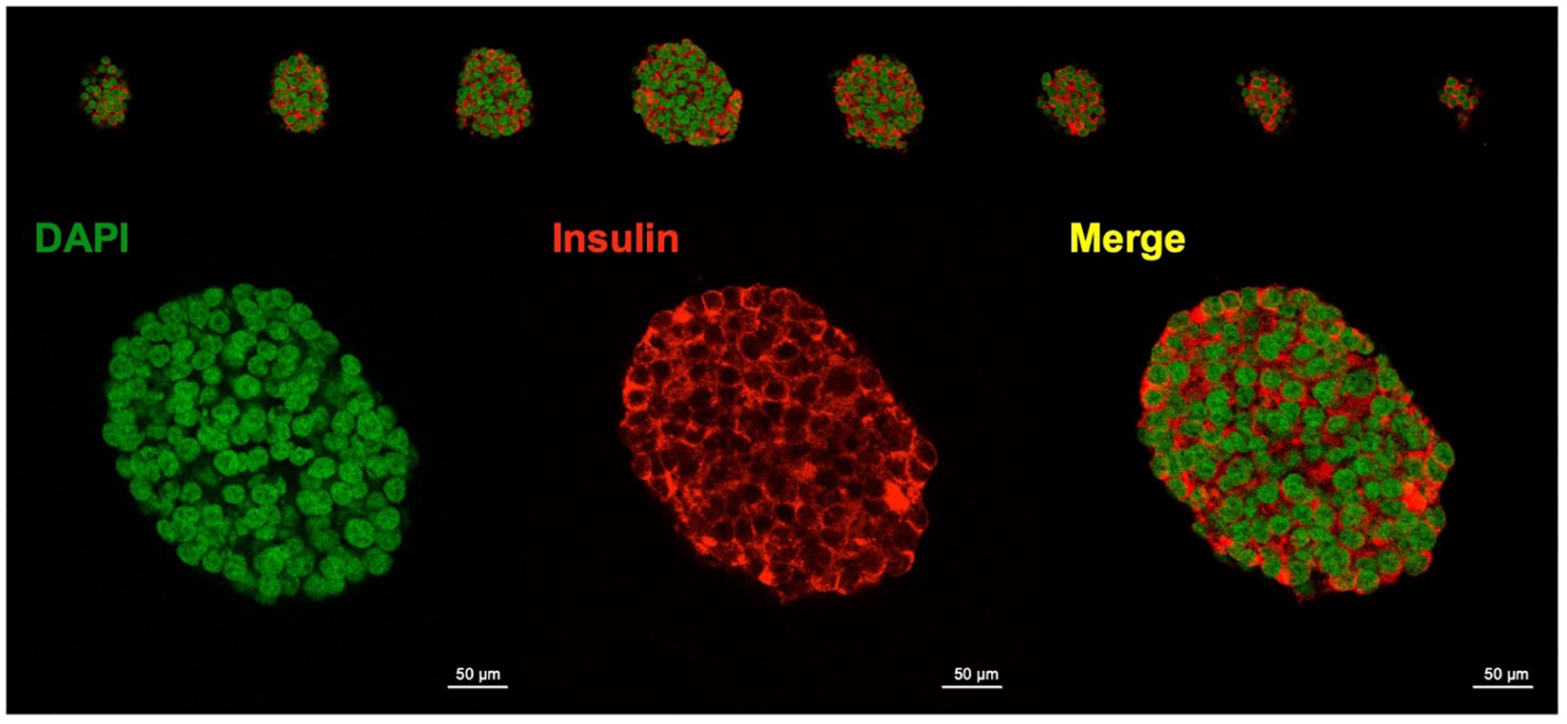
Confocal immunofluorescence of EndoC–βH1 spheroids confirms homogeneous insulin expression throughout the aggregate. Z–stack projections (top) and a single optical section (bottom) of EndoC–;βH1 spheroids at day 3 generated on agarose ultra–low–attachment dishes. Nuclei are stained with DAPI (green pseudocolour), insulin–positive dense–core granules (red), and the composite merge (yellow), indicating that essentially every cell within the aggregate retains a ;β–cell phenotype. (Scale bars = 50 μm)

To visualize insulin production within the spheroids, immunofluorescent labeling was conducted. Spheroids were centrifuged at 100 g for 5 minutes, and the pellet was fixed with 4% PFA for 15 minutes. Following washing and incubation with an anti-Insulin antibody (PA1-26938, Thermo Fisher Scientific), followed by staining with DAPI for nuclei and Alexa Fluor 488 for insulin was performed. Visualization was carried out using an 8–well chambered glass slide under a confocal microscope (**Figure 5**). Each cell within the spheroids exhibited insulin production, further demonstrating the suitability of EndoC-βH1 spheroids for subsequent experiments.

Z–stack projections (top) and a single optical section (bottom) of EndoC–βH1 spheroids at day 3 generated on agarose ultra–low–attachment dishes. Nuclei are stained with DAPI (green pseudocolor), insulin–positive dense–core granules (red), and the composite merge (yellow), indicating that essentially every cell within the aggregate retains a β–cell phenotype. (Scale bars = 50 µm).

### Glucose stimulated insulin secretion (GSIS) of β–TC–6 and EndoC–βH1 spheroids

**Figure 6:**
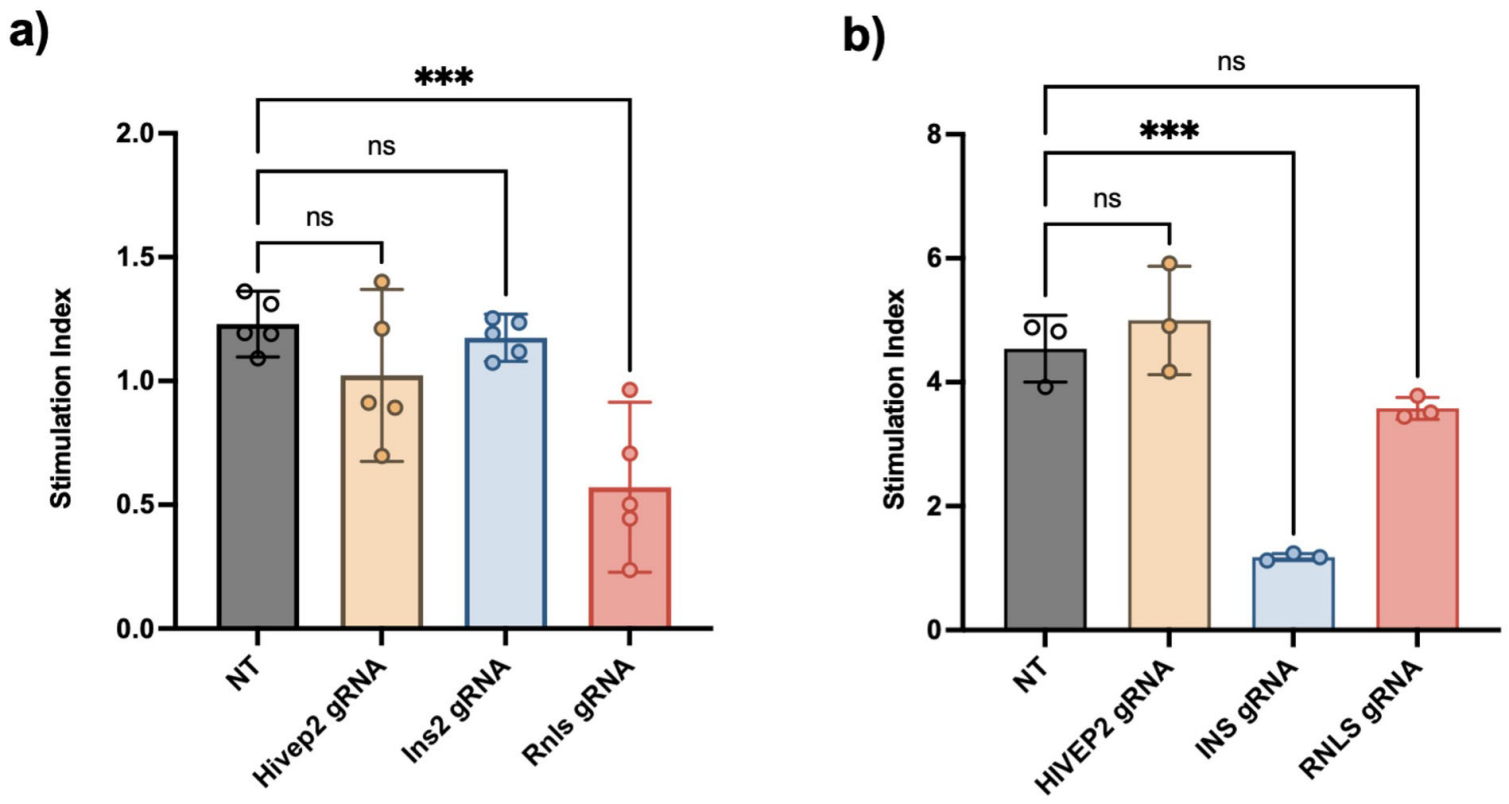
Glucose–stimulated insulin–secretion (GSIS) profiles of gene–edited β–cell spheroids. Stimulation index (ratio of insulin released at HG versus LG; mean ± SD, 30 spheroids per independent experiment) for **a**) murine β–TC–6 (n=5 independent experiment) and **b**) human EndoC–βH1 spheroids (n=3 independent experiment) carrying non–targeting (NT) or gene–specific guides. In mouse cells, cells showed no response against different glucose challenges, whereas Hivep2 deletion was neutral and Rnls deletion produced a moderate but significant reduction (P < 0.05) relative to NT.  In human cells, INS knockout again eliminated GSIS (*** P < 0.001) while neither HIVEP2 nor RNLS knockout altered the stimulation index (ns, not significant).

Glucose−stimulated insulin secretion (GSIS) was next examined to determine whether gene editing affected β–cell function (**Figure 6**). In mouse β−TC−6 cells, the NT control exhibited a stimulation index (SI) close to 1, indicating that these cells are not responsive to different glucose challenges. Disruption of *Hivep2* had no impact, yielding the SI statistically non-significant from control. Additionally, the knockout of *Ins2* resulted in the sharp decrease of insulin production. However, SI was calculated as 1 because the ratio of insulin at high glucose over low glucose is similar due to the disrupted insulin production mechanism (excluding unresponsiveness of murine β–cell). Notably, *Rnls*−edited cells showed significantly reduced SI compared to control cells, indicating that loss of *Rnls* compromises GSIS in the murine line (**Figure 6A**).

Human EndoC−βH1 cells showed the much higher SI characteristic of this more mature model. Again, *HIVEP2* deletion left glucose responsiveness intact, whereas *INS* knockout dramatically suppressed secretion. In contrast to the mouse data, *RNLS* editing produced only a modest, statistically non−significant reduction in SI, suggesting that loss of *RNLS* does not compromise insulin secretion of human β–cells (**Figure 6B**). Together, these functional assays reinforce the idea that HIVEP2 deletion is not dampen insulin secretion, while RNLS loss exerts a cell−line−dependent decrease in insulin secretion, prominent in mouse β−TC−6 cells.

Stimulation index (ratio of insulin released at HG versus LG; mean ± SD, 30 spheroids per independent experiment) for **A)** murine β–TC–6 (n=5 independent experiment) and **B)** human EndoC–βH1 spheroids (n=3 independent experiment) carrying non–targeting (NT) or gene–specific guides. In mouse cells, cells showed no response against different glucose challenges, whereas *Hivep2* deletion was neutral and *Rnls* deletion produced a moderate but significant reduction (P < 0.05) relative to NT. In human cells, *INS* knockout again eliminated GSIS (*** P < 0.001) while neither *HIVEP2* nor *RNLS* knockout altered the stimulation index (ns, not significant).

### *In vivo* transplantation of genetically modified beta cells

**Figure 7:**
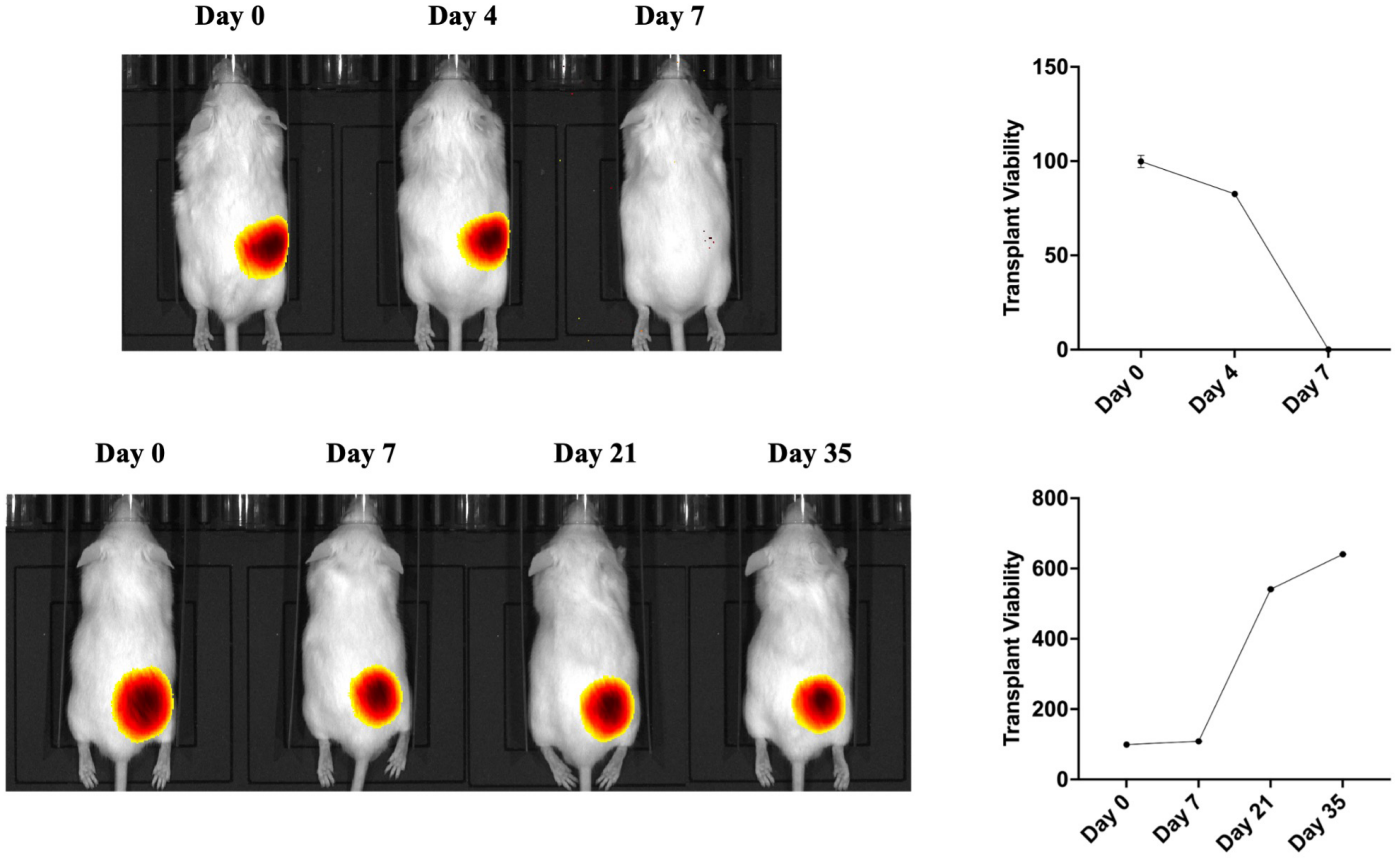
Human EndoC–βH1 graft fate in immunocompetent versus immunodeficient mice. Bioluminescence images of a CD-1 mouse (top) and a scid mouse (bottom) bearing identical luciferase-expressing EndoC–βH1 spheroids. Corresponding photon-flux curves (right) plot residual graft viability as a percentage of day-0 signals. In CD-1 mice the xenograft is rapidly rejected, with complete loss of luminescence by day 7. In contrast, the same graft in scid mice persists and progressively enlarges, demonstrating that adaptive immunity is required for xenogeneic clearance in this model.

Before initiating the gene-knockout *in vivo* transplantation experiments, a pilot experiment was done to verify whether EndoC–βH1 spheroids can survive long-term in an immunodeficient environment. Luciferase-labelled spheroids (5 × 10^6^ cells, Matrigel embedded) were implanted subcutaneously into the right flank of either CD-1 mice (fully immunocompetent) or severe combined immunodeficiency disease (SCID) mice (T and B cell deficient). Longitudinal bioluminescence imaging (d-luciferin, 150 mg/kg) revealed rapid loss of graft signal in CD-1 animals, falling below background by day 7, whereas identical grafts in scid mice persisted and expanded over a 35-day observation period (**Figure 7**). These data confirm that innate immune mechanisms alone are insufficient to eliminate the graft, and that adaptive immunity is the dominant driver of xenogeneic rejection in our system.

Bioluminescence images of a CD-1 mouse (top) and a scid mouse (bottom) bearing identical luciferase-expressing EndoC–βH1 spheroids. Corresponding photon-flux curves (right) plot residual graft viability as a percentage of day-0 signals. In CD-1 mice the xenograft is rapidly rejected, with complete loss of luminescence by day 7. In contrast, the same graft in scid mice persists and progressively enlarges, demonstrating that adaptive immunity is required for xenogeneic clearance in this model.

### Allotransplantation of β–TC–6 spheroids to CD1 mice

**Figure 8:**
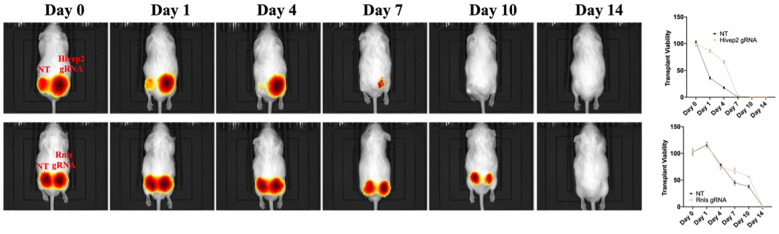
*In vivo* bioluminescence tracking of allogeneic β–TC–6 spheroid grafts in CD–1 mouse. Luciferase–expressing spheroids (5 × 10^6^ cell per flank, embedded in Matrigel) were implanted subcutaneously on day 0; left flanks carried a non–targeting (NT) control, right flanks received a gene–edited counterpart. Serial ventral images (days 0–14; identical colour scale) are shown for Hivep2 (top) and Rnls (bottom) knockouts. Line plots on the right depict residual photon flux, expressed as percentage of day–0 signal, for NT (black) versus each edit (orange). All grafts declined progressively and were undetectable by day 14; neither HIVEP2 nor RNLS deletion altered the clearance curve.

To evaluate whether targeted gene knockouts influence β–cell survival in an allogeneic transplantation context, luciferase–labelled β–TC–6 cells genetically edited for *Hivep2*, *Ins2*, and *Rnls*, as well as NT controls, were transplanted subcutaneously into the flank region of immunocompetent CD1 mice using Matrigel as a matrix (**Figure 8**). Cellular survival was assessed through bioluminescence imaging (BLI) following intraperitoneal administration of D–luciferin. The *Hivep2* knockout cells followed a survival trajectory similar to the NT controls, resulting in complete signal loss by day 7. However, a modest and transient improvement in early post-transplant protection was observed during the initial four days, as evidenced by a slightly higher residual radiance compared to controls. Despite this early trend, it remains a robust observation that single *Hivep2* deletion does not significantly extend long-term graft survival, as the protective effect was rapidly overwhelmed by the allogeneic immune response.

Interestingly, *Rnls* knockout cells exhibited prolonged survival relative to *Hivep2* knockout cells, maintaining comparable bioluminescence intensity through day 10 and exhibiting a slower decline thereafter. These findings collectively reveal that both *Rnls* and *Hivep2* did not affect β–cell survival in allogeneic transplantation, indicating that *Rnls* protective effect on autoimmune attack does not translate in allorejection, which is consistent with previously reported findings by Cai et al. (31).

Luciferase–expressing spheroids (5 × 10^6^ cell per flank, embedded in Matrigel) were implanted subcutaneously on day 0; left flanks carried a non–targeting (NT) control, right flanks received a gene–edited counterpart. Serial ventral images (days 0–14; identical color scale) are shown for *Hivep2* (top) and *Rnls* (bottom) knockouts. Line plots on the right depict residual photon flux, expressed as percentage of day–0 signal, for NT (black) versus each edit (orange). All grafts declined progressively and were undetectable by day 14; neither HIVEP2 nor RNLS deletion altered the clearance curve.

### Xenotransplantation of EndoC–βH1 spheroids to CD1 mice

**Figure 9:**
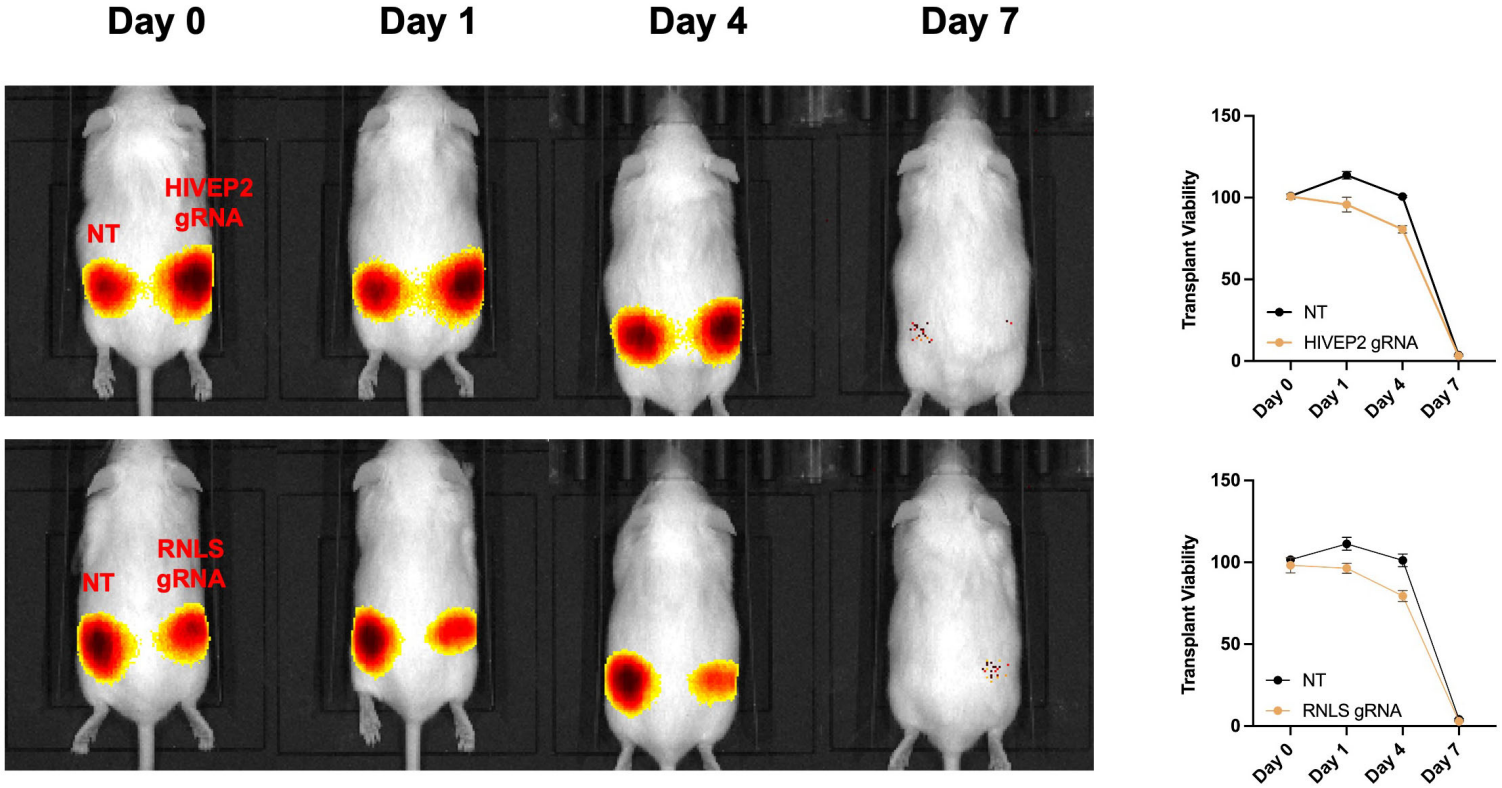
Rapid xenogeneic rejection of EndoC–βH1 spheroid grafts in CD–1 mouse. Luciferase–labelled human EndoC–βH1 spheroids (5 × 10^5^ cells per flank, Matrigel embedded) were implanted subcutaneously on day 0; the left flank received a non–targeting (NT) control, the right flank the indicated CRISPR knockout.  Sequential bioluminescence images are shown for HIVEP2 (top) and RNLS (bottom) edits, together with quantitative photon–flux decay curves (right; percentage of day–0 signal). All grafts, irrespective of genotype, lost >80 % of baseline radiance by day 4 and were completely eliminated by day 7, illustrating the innate and adaptive barriers to human–to–mouse xenotransplantation. Neither RNLS nor HIVEP2 knockout conferred any detectable survival advantage and did not alter rejection kinetics.

To test the effect of gene knockouts in xenotransplantation, luciferase−labelled EndoC–βH1 cells, harboring *HIVEP2* or *RNLS* knockouts or a NT control, were embedded in Matrigel and implanted into flank region of immunocompetent CD−1 mice (**Figure 9**). Across all groups, signal decay was markedly faster than in the allogeneic β−TC−6 transplantation, reflecting the increased severity of xenorejection. *HIVEP2*−edited grafts lost >80 % of their luminescence by day 4 and were undetectable by day 7, indicating no xenoprotective effect.

*RNLS*−edited grafts displayed a modest delay in clearance, retaining ~30 % of baseline signal on day 7 versus <10 % in controls, but were nonetheless fully rejected by day 10. Thus, in the xenogeneic CD−1 model, none of the single−gene edits conferred durable protection to human β–cells. These findings underline the greater immunological hurdle posed by xenotransplantation and suggest that more extensive engineering, potentially combining *RNLS* or *HIVEP2* loss with additional immune−evasion strategies, will be required to achieve long−term engraftment of human β–cells in fully immunocompetent hosts.

Luciferase–labelled human EndoC–βH1 spheroids (5 × 10^6^ cells per flank, Matrigel embedded) were implanted subcutaneously on day 0; the left flank received a non–targeting (NT) control, the right flank the indicated CRISPR knockout. Sequential bioluminescence images are shown for *HIVEP2* (top) and *RNLS* (bottom) edits, together with quantitative photon–flux decay curves (right; percentage of day–0 signal). All grafts, irrespective of genotype, lost >80% of baseline radiance by day 4 and were completely eliminated by day 7, illustrating the innate and adaptive barriers to human–to–mouse xenotransplantation. Neither *RNLS* nor *HIVEP2* knockout conferred any detectable survival advantage and did not alter rejection kinetics.

## Discussion

This study investigated the potential of single-gene ablation of RNLS or HIVEP2 genes, previously identified as protective against autoimmune destruction to confer immune privilege to β-cell grafts in allogeneic and xenogeneic settings. We successfully established a scalable agarose-suspension platform to generate size-controlled, high-viability murine and human β-cell spheroids with robust editing efficiency. Functional characterization revealed that while HIVEP2 deletion was functionally neutral, *Rnls* knockout modestly impaired glucose-stimulated insulin secretion in murine cells. While protein-level validation is often the standard, the high frequency of bi-allelic frame-shift indels confirmed by TIDE, combined with the observed functional impairment in GSIS for RNLS knockouts, provides a high degree of confidence in the successful disruption of the targeted loci. Importantly, *in vivo* bioluminescence tracking demonstrated that neither edit delayed graft clearance in fully immunocompetent hosts, with rejection kinetics indistinguishable from unedited controls. These data suggest that while these genes may mitigate cytokine-induced stress or specific autoimmune pathways, they do not provide a robust barrier against the multifaceted mechanisms of allo- and xenorejection.

The results obtained in this study highlight several critical insights into the complexity of immune rejection processes (39, 40). Autoimmune destruction in T1D is often a chronic, progressive process driven by specific autoreactive T-cell clones and inflammatory cytokines, pathways that RNLS (via ER stress reduction) and HIVEP2 (via NF-κB modulation) are known to buffer. In contrast, allogeneic and xenogeneic rejection involve vigorous, broad-spectrum responses including direct and indirect antigen presentation, strong innate immune activation, and, in the case of xenografts, complement-mediated lysis. Diverging from the findings of Cai et al. (31), who demonstrated that *Rnls* and *Hivep2* loss protected β-cells in non-obese diabetic (NOD) mice, our findings indicate that the resistance conferred by these genes is not potent enough to withstand this broader immune attack. This aligns with recent work (26–30) suggesting that structural concealment of major antigens (e.g., HLA class I/II knockout) or overexpression of checkpoint inhibitors (e.g., CD47, PD-L1) is required to halt allorecognition effectively.

Beyond its role in buffering inflammatory stress, recent evidence suggests that RNLS functions as a metabolic gatekeeper in β-cells. Disruption of RNLS has been shown to induce a metabolic reprogramming characterized by enhanced glycolytic flux (41). While such a shift toward aerobic glycolysis may provide a survival advantage in the autoimmune microenvironment by mitigating oxidative stress, our results suggest this intracellular metabolic adaptation is insufficient to counter the rapid, multi-effector response of allogeneic or xenogeneic rejection. Furthermore, the loss of RNLS appears to have complex systemic immunological consequences, including the modulation of natural killer (NK) cell activity (42). Given that our xenotransplantation model relies on an aggressive innate and adaptive response, the potential for metabolic reprogramming to confer protection likely meets its limit against the sheer magnitude of non-self recognition encountered in these settings.

A major strength of this work is the rigorous, head-to-head comparison across both species (mouse and human) and rejection models (allo- and xeno-) using a standardized spheroid platform. We deliberately utilized clonal cell lines (β-TC-6 and EndoC-βH1) rather than primary islets to establish a clear “proof-of-concept” baseline. Native pancreatic islets are inherently heterogeneous, varying significantly in size, cellular composition, and donor-specific immunogenicity. This biological variability acts as a confounding factor that can obscure the specific impact of a single-gene edit. By employing clonal lines, we eliminated these variables, ensuring that any difference in survival could be attributed specifically to the deletion of HIVEP2 or RNLS. Our results extend the existing literature by demonstrating that the immune-evasive limitations of RNLS are not confined to allorejection but are even more pronounced in the human-to-mouse xenogeneic setting. Furthermore, our systematic assessment of HIVEP2 establishes that this locus, despite its role in autoimmune protection, is similarly insufficient as a standalone edit for transplantation. While we observed a modest delay in early post-transplant signal loss under allogeneic conditions, this should be interpreted cautiously as hypothesis-generating finding rather than a definitive survival advantage. Such transient effects appear insufficient to overcome the robust adaptive immune barriers modeled here, necessitating more extensive engineering strategies for sustained protection. Consequently, this study serves as a critical first-step evaluation; while we acknowledge that future translational efforts must validate findings in primary human islets to capture the full complexity of the native immune response, our data provides the necessary mechanistic evidence that these single-gene targets alone are insufficient to overcome rejection, even in an optimized, uniform cellular model.

Regarding the functional status of the grafts, we observed species-specific vulnerabilities following RNLS deletion. In our murine β-TC-6 line, *Rnls* knockout resulted in a significant reduction in *Ins2* mRNA levels (**Supporting Figure 2**) and an approximately 50% decrease in the stimulation index. This downregulation and the corresponding functional impairment confirm that the loss of Rnls-mediated metabolic control compromises insulin biogenesis in this model. These results align with observations by MacDonald et al., demonstrated that while RNLS deficiency triggers a shift toward glycolysis, it can simultaneously impact metabolic coupling factors in more sensitive cell models (41). More importantly, we found that the human EndoC-βH1 line was remarkably robust to these changes, maintaining stable *INS* mRNA levels (**Supporting Figure 2**) and GSIS functionality despite successful editing. This observation directly mirrors the findings of MacDonald et al., reported that RNLS knockout in human stem cell-derived β-cells boosted basal glycolysis without significantly altering their glucose responsiveness or insulin transcript levels. The fact that rejection kinetics were indistinguishable between the functionally impaired murine cells and the metabolically active human cells strongly suggests that metabolic status was not the primary driver of the rapid graft clearance. Furthermore, TIDE analysis confirmed high-frequency bi-allelic indels (>90% in EndoC-βH1 targets), which combined with the observed murine functional phenotype, corroborates the successful disruption of the gene product across both models. However, it is important to note that the transcriptional upregulation of Hivep2 observed in our qPCR analysis (**Supporting Figure 2**) suggests a robust compensatory response. We cannot fully exclude the possibility that such large-scale transcriptional adaptations induce broader regulatory alterations or adverse indirect effects on non-targeted loci, which might further limit or negate any potential survival benefit that might otherwise be conferred by Hivep2 deletion. This cross-species comparison validates the effective disruption of RNLS and highlights the superior metabolic maturity of the human model in buffering against the metabolic rewiring induced by RNLS loss.

The implication of these findings is that stealth strategies identified in autoimmune screens cannot be assumed to function as pan-protective modifications for transplantation. Instead, these targets should be viewed as potential modifications to enhance graft fitness rather than primary drivers of immune evasion. Moving forward, long-term graft survival will certainly require a combination of strategies (24, 43). To this end, we have been developing nano-thin functional polyethylene glycol (PEG) coatings and mathematical models to investigate the effect of encapsulation parameters on the insulin secretion dynamics and viability of transplanted islets (44, 45). Furthermore, we have achieved localized immunosuppression by engineering regulatory T cell (Treg) coatings on the islet surface, demonstrating a novel strategy to create an immune-privileged microenvironment (46, 47). Our contributions complement broader advances in the field, such as HLA class I/II elimination, “don’t–eat–me” signals, or inducible suicide switches to minimize oncogenic risk (48) macro- or micro-encapsulation (49, 50), 3D bioprinting (51, 52), and local immune–modulating hydrogels (53). Together, as we recently reviewed (54), these biomaterial innovations will synergize with multi–gene editing approaches. Finally, using humanized or complement-sufficient models may provide a more predictive testing ground for these next-generation cells. By defining the limitations of single–gene edits, our study supplies a critical benchmark and an experimental framework for the layered engineering that will be required to translate β–cell replacement into a practical, immunosuppression–free cure for T1D.

## Data Availability

The original contributions presented in the study are included in the article/**Supplementary Material**. Further inquiries can be directed to the corresponding authors.
