# Supplementary material for: Single–gene knockout of RNLS or HIVEP2 are insufficient to protect β–cell spheroids from allo– and xeno–rejection
Source: Front Immunol. 2026 Feb 3;17:1759835. doi: 10.3389/fimmu.2026.1759835 (PMC12909247; doi:10.3389/fimmu.2026.1759835)
Supplement: Supplementary file 1 [file DataSheet1.docx]

**Supporting Information for**

**Single–gene knockout of RNLS or HIVEP2 are insufficient to protect β–cell spheroids from allo– and xeno–rejection**

Ismail Can Karaoglu^1^, Arda Odabas^2,3^, Tamer Onder^2,3,*^, Seda Kizilel^1,3,*^

^1^Chemical and Biological Engineering, Koc University, 34450, Sariyer, Istanbul, Turkey

^2^School of Medicine, Koç University, 34450, Sariyer, Istanbul, Turkey

^3^Research Center for Translational Medicine, Koc University, 34450, Sariyer, Istanbul, Turkey

^*^Corresponding author.

E-mail address: skizilel@ku.edu.tr

Supplementary Table 1. gRNA sequences for targeting Hivep2, Ins, and Rnls genes in both mouse and human insulin secreting beta cells.

The overhanging sequences highlighted with green for integration to pLentiCRISPRv2 plasmid after BsmBI digestion.

|  |  | 5’ – 3’ (Top oligo) | 3’ – 5’ (Bottom oligo) |
| --- | --- | --- | --- |
| **Mouse** | Hivep2_1 | CACCGCCTGTGAGAACAGAAAACGC | CGGACACTCTTGTCTTTTGCGCAAA |
|  | Hivep2_2 | CACCGTAAGGCGGATGACTCTCACA | CATTCCGCCTACTGAGAGTGTCAAA |
|  | Hivep2_3 | CACCGACAATGAGTATGAACCCGCA | CTGTTACTCATACTTGGGCGTCAAA |
|  | Ins2_1 | CACCGGTGGAACAACTGGAGCTGGG | CCACCTTGTTGACCTCGACCCCAAA |
|  | Ins2_2 | CACCGTAGAGAGCCTCTACCAGGTG | CATCTCTCGGAGATGGTCCACCAAA |
|  | Ins2_3 | CACCGCTGGGAGCCCAAACCCACCC | CGACCCTCGGGTTTGGGTGGGCAAA |
|  | Rnls_1 | CACCGCAGTAATTAGTGAACGCCAG | CGTCATTAATCACTTGCGGTCCAAA |
|  | Rnls_2 | CACCGCCACCTGAAAGATAACAAGT | CGGTGGACTTTCTATTGTTCACAAA |
|  | Rnls_3* | CACCGCTACTCCTCTCGCTATGCTC | CGATGAGGAGAGCGATACGAGCAAA |
| **Human** | HIVEP2_1 | CACCGGACAAGATGTCAGACCTAGG | CCTGTTCTACAGTCTGGATCCCAAA |
|  | HIVEP2_2 | CACCGTTCTAGGATAACCACCACTG | CAAGATCCTATTGGTGGTGACCAAA |
|  | HIVEP2_3 | CACCGGAGGTGGAAGGTAAACACAA | CCTCCACCTTCCATTTGTGTTCAAA |
|  | INS_1 | CACCGGCACAGGTGTTGGTTCACAA | CCGTGTCCACAACCAAGTGTTCAAA |
|  | INS_2 | CACCGCGGGAGGCAGAGGACCTGCA | CGCCCTCCGTCTCCTGGACGTCAAA |
|  | INS_3 | CACCGGAAGCTCTCTACCTAGTGTG | CCTTCGAGAGATGGATCACACCAAA |
|  | RNLS_1 | CACCGATTATGCCAAAAAACACCAA | CTAATACGGTTTTTTGTGGTTCAAA |
|  | RNLS_2 | CACCGTCCCACACAGCAAGGTACAA | CAGGGTGTGTCGTTCCATGTTCAAA |
|  | RNLS_3 | CACCGCATTACTTGAAAGAATCAGG | CGTAATGAACTTTCTTAGTCCCAAA |


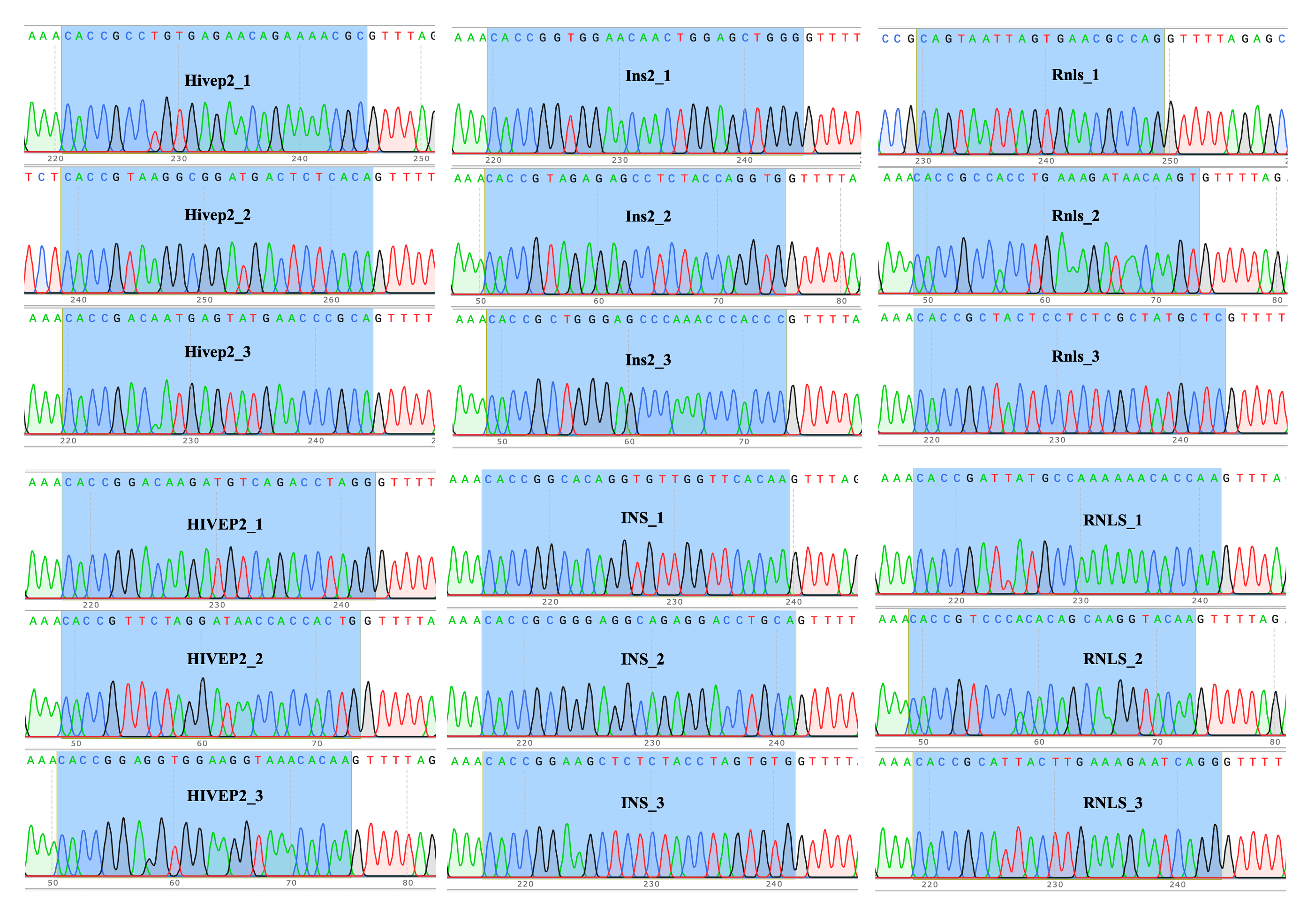


**Supplementary Figure 1. Sanger sequencing confirmation of individual gRNA inserts in the pLentiCRISPRv2 vector backbone.**

Sanger sequencing validations for gRNA cloning into lentiCRISPRv2 plasmid. (Hivep2, Ins2, Rnls for mouse cells while HIVEP2, INS, RNLS for human cells.)


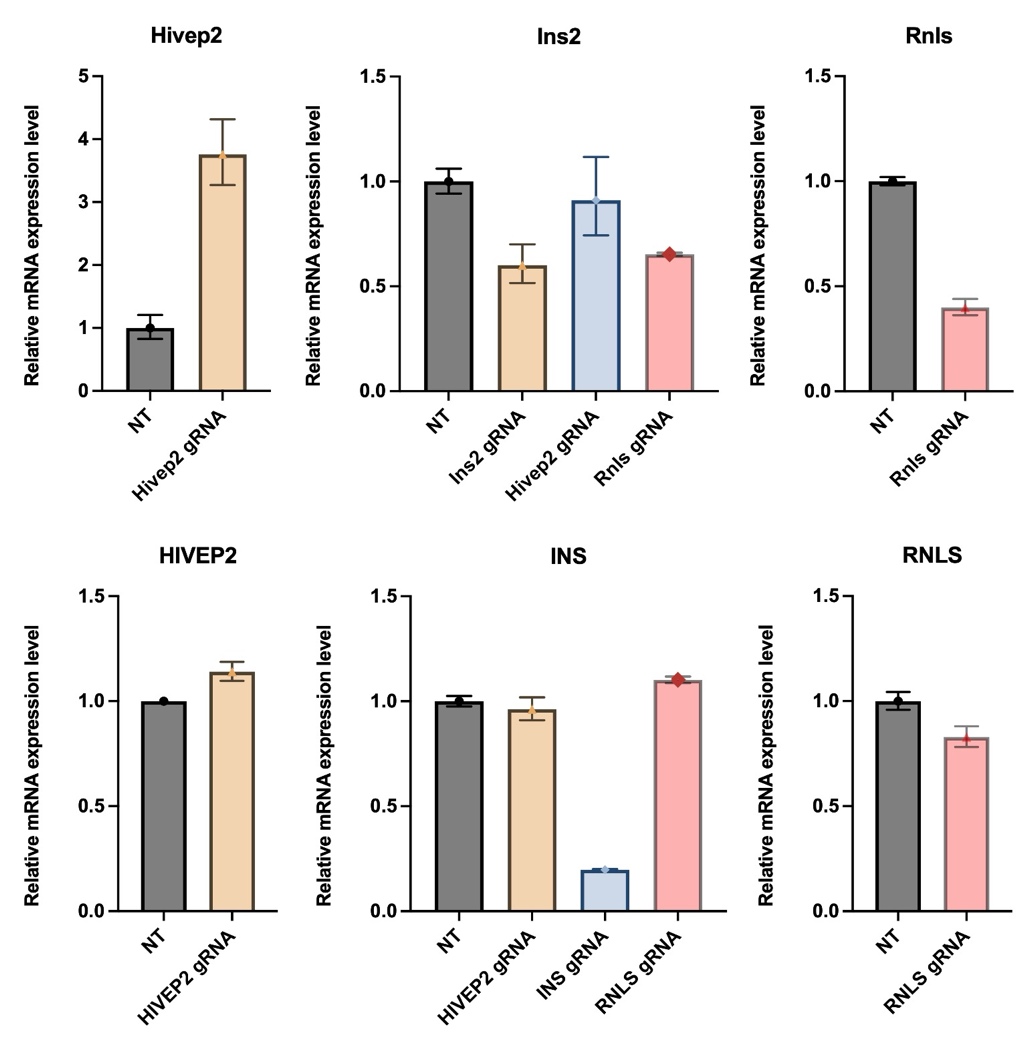


Supplementary Figure 2. Quantitative PCR analysis of target–gene transcript levels after CRISPR/Cas9 editing.

Relative mRNA abundance (2^ΔΔCt^, normalized to GAPDH) for mouse β–TC–6 spheroids and human EndoC–βH1 spheroids following transduction with the most efficient gRNA for each locus. NT, non–targeting control. Bars represent mean ± SD from three independent cultures.
